# Targeting colorectal cancer cells using AND-gated adaptor RevCAR T-cells

**DOI:** 10.3389/fimmu.2023.1302354

**Published:** 2023-12-15

**Authors:** Karla E. G. Soto, Liliana R. Loureiro, Tabea Bartsch, Claudia Arndt, Alexandra Kegler, Nicola Mitwasi, Laura Drewitz, Lydia Hoffmann, Haidy A. Saleh, Eugenia Crespo, Maria Mehnert, Cansu Daglar, Hinrich Abken, Frank Momburg, Michael Bachmann, Anja Feldmann

**Affiliations:** ^1^ Institute of Radiopharmaceutical Cancer Research, Helmholtz-Zentrum Dresden-Rossendorf, Dresden, Germany; ^2^ Mildred Scheel Early Career Center, Faculty of Medicine Carl Gustav Carus, Technical University Dresden, Dresden, Germany; ^3^ Department of Gene-Immunotherapy, Leibniz-Institute of Immunotherapy, and University Regensburg, Regensburg, Germany; ^4^ Antigen Presentation and T/NK Cell Activation Group, German Cancer Research Center, Heidelberg, Germany; ^5^ Department of Medical Oncology, National Center for Tumor Diseases (NCT), University Hospital, Heidelberg, Germany; ^6^ National Center for Tumor Diseases Dresden (NCT/UCC), partner site Dresden, Dresden, Germany; ^7^ German Cancer Research Center (DKFZ), Heidelberg, Germany; ^8^ German Cancer Consortium (DKTK), Partner Site Dresden, Dresden, Germany

**Keywords:** colorectal cancer, CAR T-cells, AND-gate targeting, CEA, EpCAM

## Abstract

Despite the success of chimeric antigen receptor (CAR) T-cells especially for treating hematological malignancies, critical drawbacks, such as “on-target, off-tumor” toxicities, need to be addressed to improve safety in translating to clinical application. This is especially true, when targeting tumor-associated antigens (TAAs) that are not exclusively expressed by solid tumors but also on hea9lthy tissues. To improve the safety profile, we developed switchable adaptor CAR systems including the RevCAR system. RevCAR T-cells are activated by cross-linking of bifunctional adaptor molecules termed target modules (RevTM). In a further development, we established a Dual-RevCAR system for an AND-gated combinatorial targeting by splitting the stimulatory and co-stimulatory signals of the RevCAR T-cells on two individual CARs. Examples of common markers for colorectal cancer (CRC) are the carcinoembryonic antigen (CEA) and the epithelial cell adhesion molecule (EpCAM), while these antigens are also expressed by healthy cells. Here we describe four novel structurally different RevTMs for targeting of CEA and EpCAM. All anti-CEA and anti-EpCAM RevTMs were validated and the simultaneous targeting of CEA^+^ and EpCAM^+^ cancer cells redirected specific *in vitro* and *in vivo* killing by Dual-RevCAR T-cells. In summary, we describe the development of CEA and EpCAM specific adaptor RevTMs for monospecific and AND-gated targeting of CRC cells via the RevCAR platform as an improved approach to increase tumor specificity and safety of CAR T-cell therapies.

## Introduction

Colorectal cancer (CRC) is one of the most common diagnosed types of cancer in developed countries (1). Non-detected and non-treated CRC can advance into alarming stages of tumor development with poor prognosis (2). Over the past years new strategies have been developed for the treatment of hematological and solid cancers (3). Among them, T-cells genetically modified to express synthetic chimeric antigen receptors (CARs) have shown impressive efficacy leading to the approval of these living drugs, particularly targeting hematological malignancies (4, 5). Unfortunately, no tumor-selecting antigen for targeting of CRC-derived cells has been detected that is either selectively expressed on CRC cells or expressed on dispensable healthy tissue (5). Nevertheless, some tumor-associated antigens (TAAs) with a high overexpression on CRC tumors and low expression on healthy tissue have been identified and could be exploited to increase the specificity of existing treatments. An example of that is the carcinoembryonic antigen (CEA, CD66e), which is normally found during oncofetal development on several tissues but during adulthood its expression is limited to the epithelia in the gastrointestinal track and other mucosal epithelial (6–8). Similarly, the epithelial cell adhesion molecule (EpCAM, CD326) is a transmembrane glycoprotein normally expressed by epithelium of healthy individuals (excepting hepatocytes and keratinocytes) (9, 10). Interestingly, an overexpression of CEA or EpCAM is usually associated with colon, stomach, pancreatic and lung cancers (6, 11–14). Although both antigens were targets in T-cell-based immunotherapy approaches, the fact that they are expressed on normal tissue represent a safety issue due to “on-target, off-tumor” toxicities (15–18). This highlights the clinical unmet need to develop more specific and controllable technologies, especially for those antigens expressed to some degree on healthy tissue.

The adaptor CAR T-cell technology constitutes a recent emerging approach that allows a better management of off-tumor effects. Compared to conventional CARs, modular (also termed adaptor) CAR T-cells do not recognize a TAA but a binding element incorporated into an adaptor molecule. This adaptor molecule serves as linker between the tumor and the adaptor CAR T-cells allowing an improved safety management. The activity of adaptor CAR T-cells can be switched on and off by the presence or absence of the adaptor molecule. By now a number of adaptor technologies have been described to target different cancer entities (19–21), including the UniCAR and RevCAR platforms developed by our group (22–36) of which the UniCAR system has shown proof of functionality in both its safety switch and efficacy in first clinical trials (NCT04230265) (37, 38). Compared to other modular CAR approaches, RevCAR T-cells lack an extracellular antibody based binding motif. Currently, they contain one of two short peptide epitopes (E5B9 or E7B6) derived from the nuclear La/SS-B protein instead (39–42). The corresponding adaptor molecules of RevCARs, called RevTMs, are bispecific antibodies (bsAbs) that recognize the respective RevCAR epitope with one binding arm and with the other one the TAA on the tumor cell surface.

Although such adaptor platforms represent a safer approach than conventional CAR T-cells, the off-tumor targeting of healthy tissue can still limit their clinical application. Thus, we and others have developed technologies that allow an improved discrimination between malignant and healthy tissues based on their protein expression pattern instead of the recognition of a single tumor related target. Pattern recognition can be achieved by integrating CARs capable of Boolean-logic signaling (e.g. OR gate, AND-gate and AND-NOT gate) (22, 43–46). Most challenging are AND-gate targeting approaches. They are based on engineering of CAR T-cells expressing a minimum of two types of receptors (signaling and co-stimulating) that recognize different TAAs. The prerequisites for a full activation of AND-gate CAR T-cells are (i) a simultaneous engagement of the two different CARs which depends on, (ii) the presence of the two different TAAs on the target cells (47). For obvious reasons, it is quite difficult to achieve a reliable simultaneous transduction of two independent CAR constructs into the same cell. The obvious disadvantage of a co-transduction is that mixtures of T-cells will occur. Only a portion of the transduced T-cells will co-express both receptor types as requested while other T-cells will only express either the signaling or the co-stimulatory gene. Moreover, the ratio of the two introduced CAR genes can vary in the respective T-cell. Alternatively, both CARs can be fused in a single viral gene construct. Due to the large size, the transduction of such a viral construct containing two CAR genes is very inefficient. In recent studies, we have consequently reduced the size of our two adaptor UniCAR genes. To achieve this goal, we replaced the extracellular scFv domain of the respective UniCAR gene by the respective epitope sequence originally recognized by the extracellular antibody domain. The resulting CAR was termed RevCAR (22). Still, the resulting RevCAR was a second-generation CAR containing both the signaling and co-stimulatory domain in the same CAR. The next step was to split the signaling and co-stimulatory domain to two separate RevCARs. The extracellular domain of the signaling (SIG) RevCAR consists of the peptide epitope E7B6, while the extracellular domain of the co-stimulatory (COS) RevCAR consists of the peptide epitope E5B9. The size of both RevCAR genes was now small enough and could be fused into a single viral vector that can be efficiently transduced into T-cells wherein both RevCAR genes are well expressed. The vector containing both RevCAR genes was termed Dual-RevCAR (22–24). As shown recently (22–24), Dual-RevCAR T-cells achieve complete activation only if the co-expressed SIG RevCARs, triggering CD3 signal, and COS RevCARs, triggering the CD28 signal are simultaneously stimulated by corresponding RevTMs (22–24). Considering that RevTMs regulate the activity of RevCAR and Dual-RevCAR T-cells, it appears most likely that the pharmacokinetics and biodistribution of the respective RevTMs directly affect the pace of the on-off switch of the modified T-cells. For the clinical application, both short and longer-living RevTMs should be available: The smaller, short-living RevTMs could be infused into the patient at early stages of the treatment, when a faster tuning of the T-cells is needed to restrain side effects, while the bigger, long-living molecules would be favorable at later stages to avoid continuous infusion of RevTMs (32, 48).

Here we describe the development of novel RevCAR TMs for combinatorial targeting of CEA and EpCAM expressing cells. The anti-CEA RevTMs are structurally based on an either scFv or IgG4-backbone while the additional anti-EpCAM RevTMs are based on an either minibody (Mb) or IgG1-format. The combination of these novel anti-CEA and anti-EpCAM RevTMs with the Dual-RevCAR T cell platform allowed us to achieve a true AND-gate combinatorial targeting of CRC cells.

## Materials and methods

### Cell lines and cell culture conditions

3T3 and HEK-293T were obtained from ATCC (Manassas, VA, USA), LS174T and HT-29 were purchased from DSMZ (Braunschweig, Germany) without additional authentication, while MDA-MB-231 were obtained as described before (49). The expression of human CEA on HT-29 cells is low, but for our purposes we genetically modified them to overexpress this antigen and designated them as HT-29 CEA^HIGH^. Additionally, for *in vivo* studies the cells were genetically engineered to express the firefly Luciferase (Luc^+^) via lentiviral transduction and designated HT-29 CEA^HIGH^ Luc^+^. The cell lines 3T3, HEK-293T and MDA-MB-231 were maintained in DMEM complete, whereas LS174T, HT-29 CEA^HIGH^ and HT-29 CEA^HIGH^ Luc^+^ were cultured in RPMI complete (50). All cells were periodically assessed for mycoplasma and kept at 37°C in a humidified atmosphere of 5% CO_2_.

### Spheroid formation

To form spheroids, HT-29 CEA^HIGH^ Luc^+^ cells (9,000 cells per well) were seeded in a transparent F-bottom or white chimney well F-bottom CELLSTAR plate (Greiner Bio-One, Frickenhausen, Germany) coated with 1% agarose and centrifuged at 850 ×g for 3 min. After 3 days of incubation, spheroid formation was confirmed and posterior assays were performed. In order to confirm cell number, spheroids were collected individually and incubated in trypsin/EDTA for 10 min at 37°C with shaking. Dissociated cells were then counted using trypan blue and Neubauer chamber.

### Isolation of human peripheral blood mononuclear cells and transduction of human T-cells

Buffy coats of healthy voluntary donors were acquired from the German Red Cross (Dresden, Germany) after written consent. Human PBMCs were isolated with Pancoll (Aidenbach, Germany) via gradient centrifugation using LeucoSEP™ tubes (Greiner Bio-One). Then, primary T-cells were obtained using the Pan T-cell Isolation Kit (Miltenyi Biotec, Bergisch Gladbach, Germany) following the supplier’s instructions. To produce RevCAR and Dual-RevCAR T-cells, isolated T-cells were transduced with the respective lentivirus as previously described (22).

### Design and purification of RevTMs

Four structurally different molecules specific to detect CEA or EpCAM were designed following the general structure of a RevTM (22). The two RevTMs recognizing CEA integrate an scFv able to bind membrane-bound human CEA (51) and an scFv that recognizes the E7B6 epitope on the RevCAR T-cells. Additionally, one of the molecules includes an IgG4-derived backbone incorporating mutations. In addition, two structurally different molecules recognizing EpCAM were designed by fusing an scFv derived from the anti-EpCAM mAb HEA125 (52, 53) and an scFv recognizing the E5B9 RevCAR epitope. One of the molecules included the hinge, C_H_2 and C_H_3 domains of a human IgG1 (E216-K447) harboring mutations abrogating FcγRIII binding as previously reported (54); while the other RevTM bound the two scFvs through a hinge and C_H_3 domain. Each molecule was further cloned into a lentiviral vector and 3T3 cells were transduced to produce the respective RevTM (55). All vectors included a signal peptide (SP) allowing the secretion of the RevTMs into the supernatant, which facilitates their purification. Anti-CEA RevTMs include a 6xhistidine (His) Tag that allows their purification via Ni-NTA chromatography (QIAGEN, Hilden, Germany), while anti-EpCAM molecules incorporate StrepTag-II sequences which facilitate their purification through anti-Strep-Tactin^®^ XT gravity columns (Iba-Lifesciences, Göttingen, Germany). To assess the yield and purity, the molecules were separated via SDS-PAGE followed by staining with Quick Coomassie solution (56) and immunoblotting. After transferring the proteins to a nitrocellulose membrane, separated anti-CEA RevTMs were detected as previously reported (57), whereas anti-EpCAM RevTMS were detected by an anti-human-IgG-HRP mAb (Merck, Darmstadt, Germany, #A6029) and the Amersham ECL Prime Western Blotting Detection Reagent kit (Cytiva Europe, Freiburg, Germany). The signal was exposed and measured with a ChemiDoc XRS+ system (Bio-Rad Laboratories, Feldkirchen, Germany).

### Flow cytometry analysis

To analyze the binding of RevTMs to cancer cells and RevCAR T-cells, flow cytometry was performed as detailed before (22). To demonstrate the expression of E5B9 and E7B6 epitopes on the modified RevCAR-T cells, in-house produced anti-5B9 and anti-7B6 mAbs were included. For detection of RevTM binding to T-cells and target cancer cells PE-conjugated anti-His (Miltenyi Biotec, #130120718) and DY-649-conjugated anti-Strep-Tactin^®^ XT (Iba-Lifesciences, #2-1568-050) together with PE-conjugated anti-mouse IgG (BioLegend, Koblenz, Germany, #405307) were used. Additionally, commercial mAbs recognizing CEA and EpCAM (Cell Signaling Technology, Danvers, MA, USA, #2383 and #2929) together with an AlexaFluor647-conjugated anti-mouse IgG mAb (Invitrogen, Karlsruhe, Deutschland, #A-21235) were employed to evaluate the expression of the antigens on the cell surface. As negative control, a mouse IgG1 isotype control (BD Biosciences, Heidelberg, Germany, #554121) was included. Furthermore, the antigen density of cancer cells was assessed with the QIFIKIT (Agilent, Waldbronn, Germany) and PE-conjugated anti-mouse IgG mAb (BioLegend, #405307) as explained before (22). All samples were analyzed using a MACSQuant^®^ Analyzer and MACSQuantify Software (Miltenyi Biotec).

### Activation phenotyping of modified T-cells

In order to study their activation status genetically modified T-cells (5x10^4^) were cultured together with cancer cells at a 5:1 effector to target cell (E:T) ratio in the absence, presence or combination of RevTMs. After 48 h incubation, all cells were collected and stained for 20 min at 4°C in the dark with APC-conjugated anti-CD69 mAb (Miltenyi Biotec, #130-112-614).

### *In vitro* cytotoxicity assays

The tumor cell lysis mediated by RevCAR and Dual-RevCAR T-cells was evaluated *in vitro* by standard chromium (^51^Cr)-release (55) and luciferase-based assays. To assess the killing capacity of RevCAR T-cells, triplets of ^51^Cr-labelled cancer target cells (5x10^3^) were cultured simultaneously with RevCAR T-cells in the presence or absence of anti-CEA RevTMs at different E:T ratios (5:1, 2:1, 1:1 and 1:2) for 24 h. In a similar manner, different concentrations of each RevTM were tested to estimate EC_50_ values. Following a similar experimental set-up, ^51^Cr-labelled cancer cells were cultured together with Dual-RevCAR T-cells in a 5:1 E:T ratio under different conditions: in the (I) absence, (II) presence or (III) combination of RevTMs for 48 h. A luciferase-based killing assay was performed to assess the killing of spheroids. Briefly, HT-29 CEA^HIGH^ Luc^+^ spheroids seeded in a white chimney well F-bottom CELLSTAR plate were co-cultured with Dual-RevCAR T-cells at 1:1 E:T ratio under the same conditions as the monolayer assays. After 20 h incubation, ONE-Glo™ reagent was added to each sample and incubated 5 min in the dark. The luminescence signal was then measured and analyzed using the Infinite M200 pro and i-control™ (TECAN, Crailsheim, Germany).

### Cytokine release assay

To estimate the abundance of secreted cytokines, cell-free supernatants from a co-culture of RevCAR or Dual-RevCAR T-cells and cancer cells in the absence, presence or combination of RevTMs were harvested after 24 h or 48 h incubation. Supernatants were then analyzed using the BD OptEIA™ Human IFN-γ, IL-2, TNF-α and GM-CSF ELISA Sets (BD Biosciences) or the MACSPlex Cytokine 12 human kit (Miltenyi Biotec) according to manufacturer’s instructions. Values below the detection level were set to 0.

### Tumor xenograft models

Eight-week old female NXG mice (Janvier Labs, Le Genest-Saint-Isle, France) were kept in a pathogen free facility with 12 h light/dark cycle and they were daily supervised by husbandry personnel. The mice were randomly designated to experimental groups of five animals. To evaluate *in vivo* performance of RevCAR T-cells, HT-29 CEA^HIGH^ Luc^+^ cells (1x10^6^) were injected subcutaneously with or without RevCAR T-cells (0.5x10^6^) and RevTM (200 pmol) in a final volume of 100 µl PBS. Following a similar experimental set-up, the performance of Dual-RevCAR T-cells *in vivo* was assessed by injecting subcutaneously HT-29 CEA^HIGH^ Luc^+^ cells (1x10^6^) without or with Dual-RevCAR T-cells (1x10^6^) in the absence, presence or combination of RevTMs (100 pmol) in a final volume of 100 µl PBS. To measure the luciferase-activity of tumor cells, anesthetized mice were subcutaneously injected with 150 µl D-Luciferin and optically measured with the InVivo Xtreme (Bruker, Nehren, Germany) or the IVIS Spectrum *In Vivo* Imaging System (PerkinElmer, Rodgau, Germany) at several time points as previously described (36). All animal procedures have been approved by the local ethics committee for animal experiments and were performed at the Helmholtz-Zentrum Dresden-Rossendorf (HZDR) in compliance with the German regulations of animal welfare.

### Statistical analysis

Statistical analysis was performed using GraphPad Prism 9 (GraphPad Software Inc., La Jolla, CA, USA) as indicated in the respective figure legend. P values below 0.05 were considered significant: p < 0.05 (*), p < 0.01 (**), p < 0.001 (***).

## Results

### Design, expression and purification of anti-CEA RevTMs

**Figure 1:**
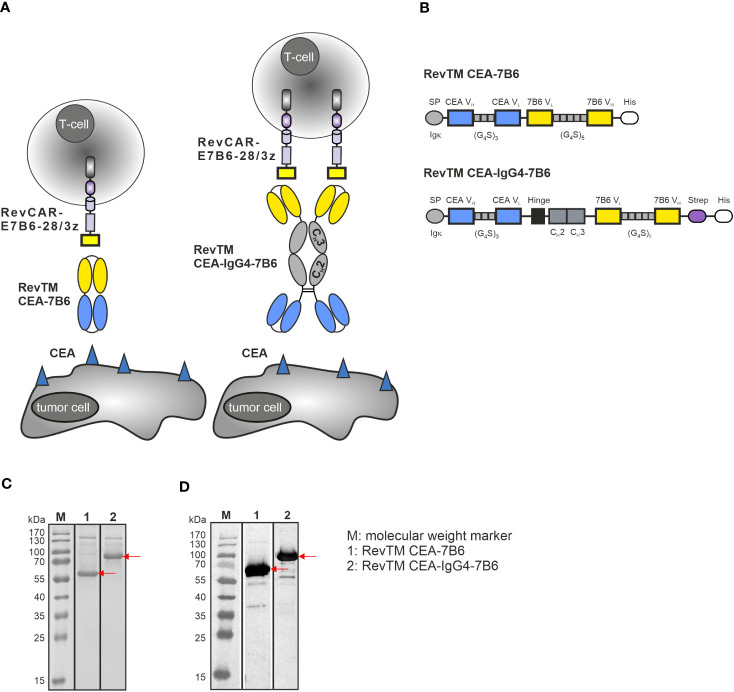
RevCAR system and novel anti-CEA RevTM constructs. **(A)** For redirection of RevCAR T-cells to CEA-expressing cancer cells, CEA-specific RevTMs of two different formats were generated: The RevTM CEA-7B6 is based on an scFv, while the RevTM CEA-IgG4-7B6 on an IgG4 backbone. **(B)** Both RevTMs are bispecific antibodies (bsAbs) that consist of a CEA-specific scFv derived from the variable heavy (V_H_) and light chain (V_L_) domains of an anti-CEA mAb. The anti-CEA binding arm is connected via a glycine (G)-serine (S) linker to a second scFv directed against the E7B6 epitope expressed in the extracellular domain of the RevCAR. The RevTM based on an IgG4 backbone includes the hinge, C_H_2 and C_H_3 domains of a mutated human IgG4 molecule. Due to the cysteine residues present in the hinge region, disulfide bridges promote the arrangement of homodimers. All RevTMs contain an Igk signal peptide (SP) for their secretion and a 6x-histidine (His) tag for their purification and detection. After their expression in transduced 3T3 cells, RevTMs (indicated by the arrows) were purified from cell supernatants via Ni-NTA columns, separated by SDS-PAGE and then analyzed using **(C)** Quick Coomassie staining and **(D)** immunodetection with anti-His Ab on a nitrocellulose membrane.

To redirect RevCAR-E7B6 T-cells against CEA-expressing tumor cells, we generated two anti-CEA-anti-7B6 RevTMs of different formats and sizes (**Figure 1A**). Therefore, the sequence of an anti-CEA scFv was fused to the sequence of the anti-7B6 scFv either directly, resulting in the scFv-based RevTM CEA-7B6 or via a human IgG4 Fc backbone, resulting in the IgG4-based RevTM CEA-IgG4-7B6. The IgG4 Fc backbone of the latter RevTM consists of the hinge, IgG4 C_H_2 and C_H_3 domain mutations in the hinge and C_H_2 regions to reduce Fcγ receptor binding. In contrast to the scFv-based format, the RevTM CEA-IgG4-7B6 forms homodimers due to disulfide bonds between the cysteine residues in the hinge region as schematically shown in **Figure 1A**. All RevTMs contain the N-terminal signal peptide of murine Igκ to mediate secretion and the C-terminal His tag to allow the purification and detection of the RevTMs (**Figure 1B**).

For expression, RevTM sequences were cloned into lentiviral vectors and transduced in eukaryotic 3T3 cell lines. RevTMs were purified by Ni-NTA affinity chromatography from the culture supernatants. Afterwards, eluted fractions were separated by SDS-PAGE and analyzed by Quick Coomassie staining (**Figure 1C**) or blotted on a nitrocellulose membrane and detected using an anti-His Ab (**Figure 1D**). As shown in **Figures 1C, D**, although RevTM CEA-7B6 and RevTM CEA-IgG4-7B6 have a theoretical size of around 57 kDa and 83 kDa (respectively, monomeric under reducing conditions), both RevTMs run slightly higher on SDS-PAGE. Besides the dominant protein bands reflecting the full-length RevTMs, some minor high molecular weight bands were observed, which might be non-His-tagged recombinant proteins from the cell culture media or aggregates of the RevTMs. Both RevTMs CEA-7B6 and CEA-IgG4-7B6 were successfully expressed and purified in sufficient yield and quality for pre-clinical functionality studies.

### Characterization of binding capability of novel anti-CEA RevTMs

**Figure 2:**
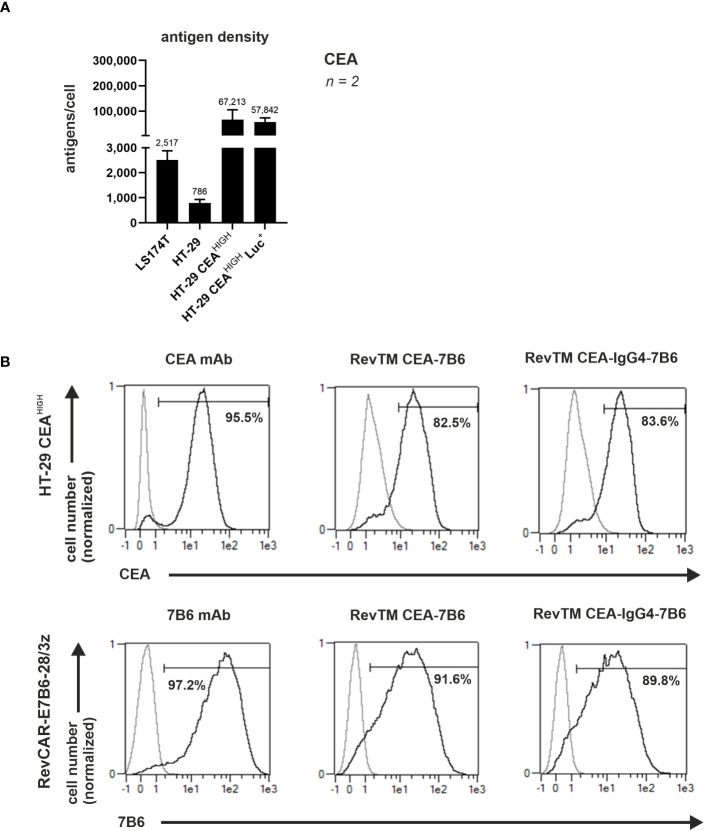
Binding of anti-CEA RevTMs to tumor cells and RevCAR T-cells. **(A)** CEA density on the surface of target cells was assessed and quantified. Data for two individual experiments are summarized as mean ± SD. Calculated mean value is shown above each column. **(A)** HT-29 CEA^HIGH^ cells and **(B)** RevCAR-E7B6-28/3z T-cells were stained with the novel anti-CEA RevTMs and detected with PE-conjugated anti-His mAb. Stainings with CEA and 7B6 mAbs served as positive controls. The commercially available CEA mAb was detected with AlexaFluor647-conjugated anti-mouse IgG mAb and the 7B6 mAb with PE-conjugated anti-mouse IgG mAb. Stained cells (black curves) and corresponding controls (gray curves) are displayed as histograms and the percentage of positively stained cells is indicated. Results for one representative binding assay are shown.

To assess the binding capability and functionality of the novel anti-CEA RevTMs, the human colon cancer cell lines LS174T and HT-29 were used. Since CEA expression on HT-29 cells was low, we genetically modified this cell line to recombinantly overexpress CEA by lentiviral transduction. The resulting cell line was named HT-29 CEA^HIGH^. As determined by flow cytometry, we estimated 67,213 molecules of CEA/cell for the artificially CEA overexpressing cell line HT-29 CEA^HIGH^ (**Figure 2A**). Using the same approach, we estimated only 2,517 molecules of CEA/cell for the native CEA positive LS174T cells. Although LS174T cells are commonly accepted as CEA high-expressing cell model, based on our estimated expression levels, we employed here the HT-29 CEA cells as a CEA^HIGH^-expressing cell model and the LS174T cells as a CEA^LOW^-expressing model.

In order to cross-link CEA^+^ tumor cells with RevCAR-E7B6-armed T-cells, the bispecific anti-CEA-anti-E7B6 RevTMs must recognize CEA on tumor cells and the E7B6 epitope on RevCAR-modified T-cells. To confirm both binding capabilities of the novel anti-CEA-anti-E7B6 RevTMs flow cytometry was used. As shown in **Figure 2B**, both anti-CEA RevTMs bound to HT-29 CEA^HIGH^ cells and to RevCAR-E7B6 T-cells proving both binding specificities. To estimate binding affinity towards HT-29 CEA^HIGH^ cells a titration of the novel RevTMs was performed. As shown in **Supplementary Figure 1**, the apparent binding affinity for RevTM CEA-7B6 was lower (K_D_ = 175.2 nM) in comparison to the RevTM CEA-IgG4-7B6 (K_D_ = 35.6 nM). Considering the homodimer formation of the RevTM CEA-IgG4-7B6 resulting in two binding arms towards CEA, it most likely binds more efficiently to CEA-expressing tumor cells than the monovalent RevTM CEA-7B6. Overall, these results demonstrate that the novel molecules are able to specifically bind both, the target of interest as well as to RevCAR-E7B6 T-cells.

### *In vitro* cytotoxic activity and cytokine release of RevCAR T-cells against CEA-expressing cells redirected by the anti-CEA RevTMs

**Figure 3:**
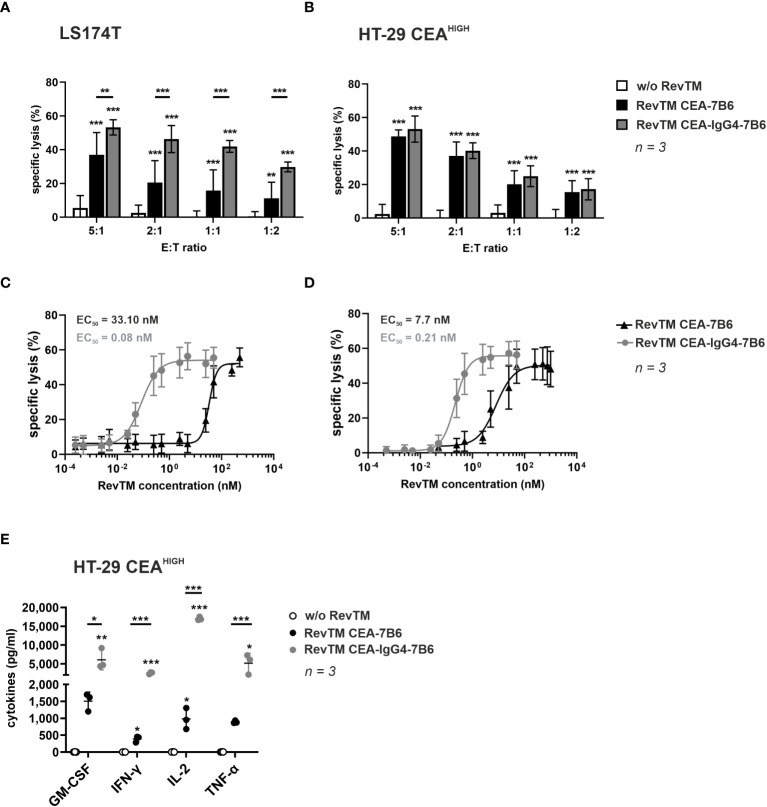
*In vitro* killing potential and cytokine secretion of RevCAR T-cells redirected to CEA-expressing tumor cells. To assess cytotoxic activity, ^51^Cr-labeled **(A, C)** LS174T or **(B, D)** HT-29 CEA^HIGH^ cells were cultured together with RevCAR-E7B6-28/3z T-cells in the absence or presence of RevTM CEA-7B6 or RevTM CEA-IgG4-7B6 **(A, B)** either at a saturating RevTM concentration at different E:T ratios or **(C, D)** with different RevTM concentrations at 5:1 E:T ratio. Data for three individual experiments are shown as mean ± SD. **(E)** To evaluate cytokine production, RevCAR-E7B6-28/3z T-cells were cultured together with HT-29 CEA^HIGH^ cells in the absence or presence of RevTMs in a saturating concentration at a 5:1 E:T ratio. After 24 h incubation, cytokine level concentrations in the supernatants were determined. Results for individual T-cell donors are shown as individual points (mean ± SD). Statistical significance of experimental groups against control group was determined by one-way ANOVA with Dunnett’s multiple comparison test, whereas significant difference between experimental groups was evaluated with one-way ANOVA followed by Tukey’s test; p < 0.05 (*), p < 0.01 (**), p < 0.001 (***).

The ability of the novel anti-CEA RevTMs to promote specific killing of target cells was recorded by chromium-51 (^51^Cr)-release assays. For this, RevCAR T-cells were incubated together with LS174T or HT-29 CEA^HIGH^ cells at different E:T ratios in the absence or presence of RevTM CEA-7B6 or CEA-IgG4-7B6. As demonstrated in **Figures 3A, B**, both anti-CEA RevTMs successfully redirected RevCAR T-cells to kill CEA^+^ cells even at low E:T ratios. Interestingly, RevTM CEA-7B6 promoted a lower killing of LS174T cells compared to RevTM CEA-IgG4-7B6, indicating higher sensitivity of RevTM CEA-IgG4-7B6 to CEA at lower levels. Nonetheless, both of the RevTMs endorsed a similar cytotoxic activity against HT-29 CEA^HIGH^ cells. Moreover, no killing was observed in the absence of RevTMs, reflecting that the modified T-cells will only be specifically activated and promote cell killing in the presence of RevTMs. Using a similar assay set up, RevCAR T-cells were co-cultured together with LS174T or HT-29 CEA^HIGH^ cells with increasing concentrations of the anti-CEA RevTMs in order to calculate the half-maximal effective concentration (EC_50_) values. As displayed on **Figure 3C, D**, the RevTM CEA-IgG4-7B6 (EC_50_ values in the picomolar range) was more efficient compared to the RevTM CEA-7B6 (EC_50_ values in the nanomolar range) suggesting that the increased valency and avidity of the IgG4-based RevTM improves the killing efficiency of the redirected RevCAR T-cells.

After validation of the specific cytotoxic potential of RevCAR T-cells promoted by the novel anti-CEA RevTMs, the cytokine release pattern was evaluated. For this, RevCAR T-cells were co-cultured with HT-29 CEA^HIGH^ cells in the presence or absence of RevTM CEA-7B6 or CEA-IgG4-7B6. After 24 h incubation, cell-free supernatant was analyzed for cytokine release by ELISA. As summarized in **Figure 3E**, RevCAR T-cells secreted GM-CSF, IFN-γ, IL-2 and TNF-α upon engaging CEA^+^ cells via anti-CEA RevTMs. Interestingly and, in line with the results from the killing assays, RevTM CEA-IgG4-7B6 induced a higher cytokine release compared to the RevTM CEA-7B6. No increase in cytokine levels were detected when the RevCAR T-cells were co-cultured with the target cancer cells in the absence of RevTM showing that the cytokine release from RevCAR T-cells occurs in a RevTM-dependent manner.

Overall, these results indicate that the novel anti-CEA RevTMs successfully redirect RevCAR T-cells to effectively kill CEA-expressing colon cancer cell lines and to promote secretion of proinflammatory cytokines.

### *In vivo* inhibition of tumor growth by RevCAR T-cells induced by anti-CEA RevTMs

**Figure 4:**
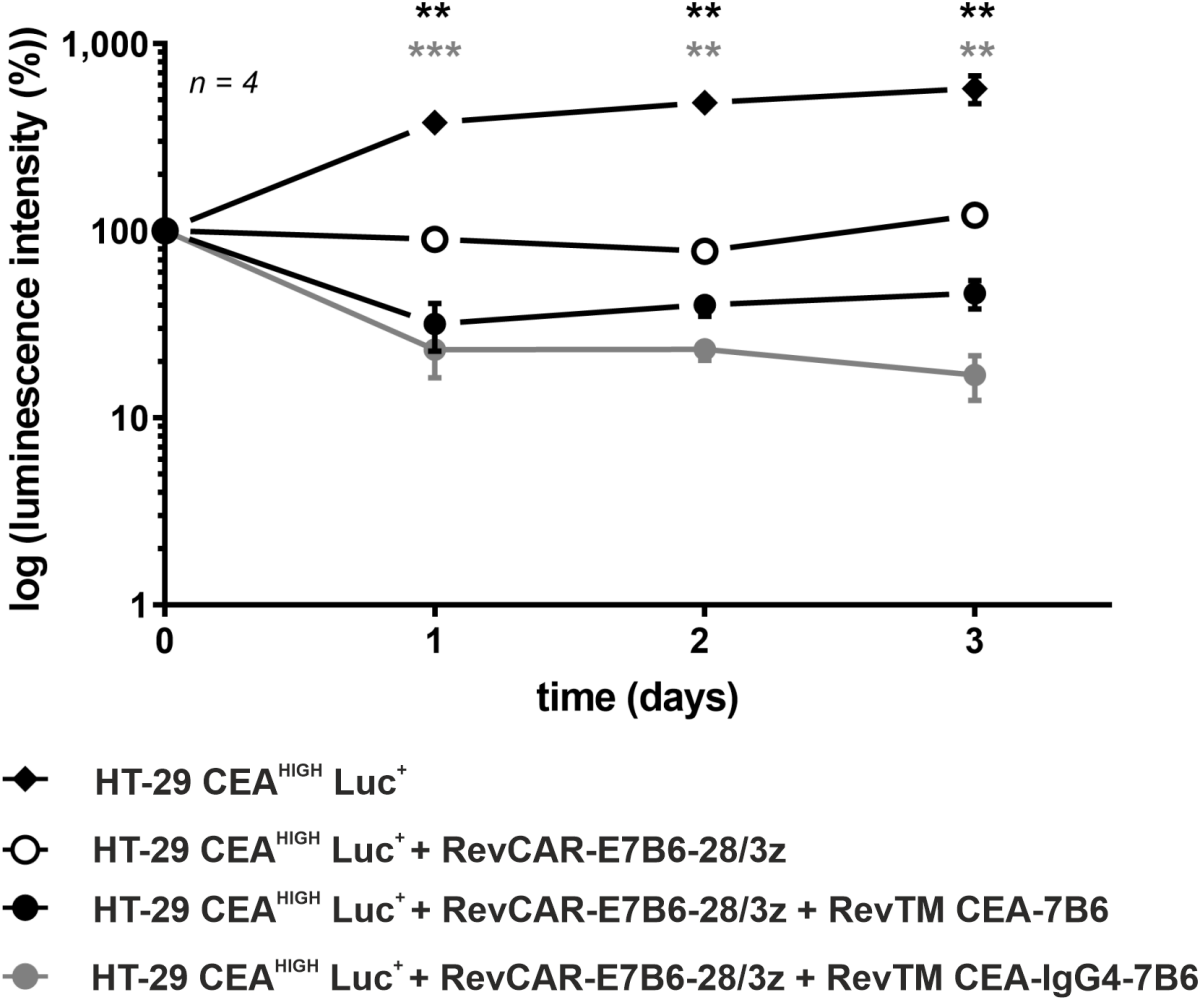
*In vivo* killing of HT-29 CEA^HIGH^ Luc^+^ cells by redirected RevCAR T-cells. Female NXG mice were injected with luciferase (Luc)-expressing HT-29 CEA^HIGH^ Luc^+^ cells alone, together with RevCAR-E7B6-28/3z T-cells in the absence or presence of the RevTMs CEA-7B6 or CEA-IgG4-7B6. A quantitative analysis was performed and the values were normalized to the initial measurement performed after injection on day 0 and represented as mean of log luminescence intensity ± SD. For statistical analysis two-way ANOVA with Dunnett’s comparison test was performed. Significant differences between control group HT-29 CEA^HIGH^ Luc^+^ + RevCAR-E7B6-28/3z and the experimental groups including RevTM CEA-7B6 or RevTM CEA-IgG4-7B6 are showed with asterisk (*, black or gray respectively); p < 0.01 (**), p < 0.001 (***).

The ability of the anti-CEA RevTMs to promote cytotoxic activity of the RevCAR T-cells *in vivo* was studied. For ethical reasons to minimize the number of experimental mice, only HT-29 CEA^HIGH^ cells were used for the *in vivo* proof-of-concept study. In order to visualize the tumor growth in mice, HT-29 CEA^HIGH^ cells were further modified to express firefly luciferase (HT-29 CEA^HIGH^ Luc^+^). Control groups received tumor cells alone or in combination with RevCAR T-cells. The treated groups received tumor cells, RevCAR T-cells together with either RevTM CEA-7B6 or RevTM CEA-IgG4-7B6. Tumor growth was monitored by bioluminescence imaging. As summarized in **Figure 4**, treatment of mice with RevTM CEA-7B6 or RevTM CEA-IgG4-7B6 led to potent inhibition of tumor growth in contrast to the control groups. In accordance with the *in vitro* data, the administration of RevTM CEA-IgG4-7B6 caused a stronger restriction of the tumor growth in comparison to RevTM CEA-7B6. Taken together, data demonstrate an effective and RevTM-dependent growth inhibition of CEA^+^ tumors by redirected RevCAR T-cells using anti-CEA RevTMs.

### Dual-RevCAR system for combinatorial AND-gate targeting of CEA and EpCAM

Besides overexpression on cancer cells, CEA is also present on healthy tissues, which can cause off-tumor effects. To increase tumor selectivity, we aimed to establish a combinatorial Dual-RevCAR targeting approach following an AND-gate logic. Therefore, EpCAM was chosen as additional tumor target in combination with CEA since both TAAs are known to be co-expressed on colorectal cancer cells.

**Figure 5:**
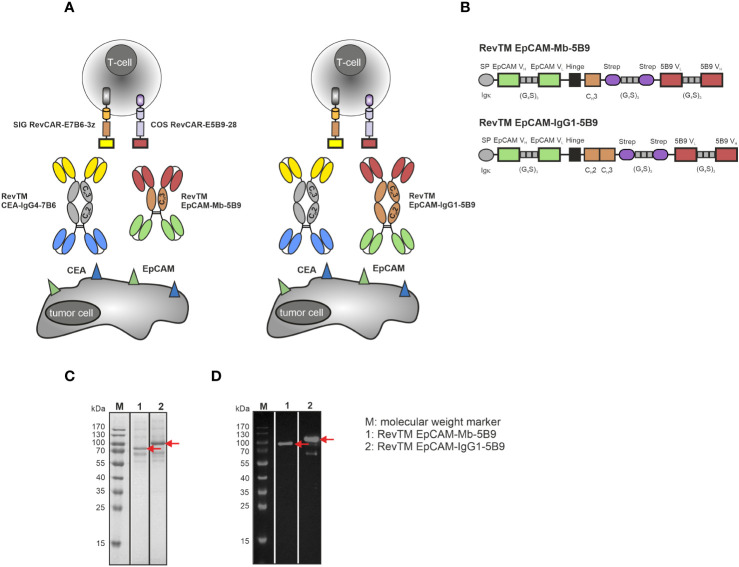
Dual-RevCAR system for combinatorial AND-gate targeting of CEA and EpCAM with anti-CEA and anti-EpCAM RevTMs. **(A)** Dual-RevCAR T-cells are modified to express two separate receptors: the signaling (SIG) RevCAR-E7B6 triggering the CD3 signal and the co-stimulatory (COS) RevCAR-E5B9 triggering the CD28 co-stimulatory signal. The Dual-RevCAR T-cells are redirected to CEA^+^ EpCAM^+^ cancer cells via the RevTM CEA-IgG4-7B6 and newly generated anti-EpCAM-anti-5B9 RevTMs. The simultaneous recognition of CEA and EpCAM by SIG and COS RevCARs via the respective RevTMs triggers both CD3 and CD28 signals resulting in complete activation of Dual-RevCAR T-cells according to a combinatorial AND-gate logic. **(B)** The anti-EpCAM RevTMs consist of an EpCAM-specific scFv derived from the variable heavy (V_H_) and light chain (V_L_) domains of an anti-EpCAM mAb. This scFv is connected via glycine (G)-serine (S) linkers to a second scFv directed against the RevCAR E5B9. The minibody (Mb)-based RevTM EpCAM-Mb-5B9 connects the two scFvs via hinge-C_H_3, whereas the RevTM EpCAM-IgG1-5B9 through hinge-C_H_2-C_H_3 domains from a human IgG1. The latter also comprises mutations to silence the Fc region. Due to the cysteine residues present in the hinge region, disulfide bridges form causing the generation of homodimers. The RevTMs contain an Igk signal peptide (SP) that allows their secretion into the cell supernatant and StrepTag-II sequences, which facilitate purification and detection. Anti-EpCAM RevTMs (indicated by red arrows) were purified from cell supernatants via anti-Strep-Tactin^®^ XT columns, separated by SDS-PAGE and then analyzed using **(C)** Quick Coomassie staining and **(D)** immunodetection with anti-human-IgG-HRP mAb on a nitrocellulose membrane.

To achieve a true dual targeting according to an AND-gate logic, in the Dual-RevCAR T-cells the CD3 activation signal (SIG) and CD28 co-stimulatory signal (COS) were split onto two different RevCARs being co-expressed in one immune effector cell, as shown in **Figure 5A**. As previously published (22), the SIG RevCAR consists of the extracellular peptide epitope E7B6, IgG4 hinge domain (HiD), CD4 transmembrane domain (TMD) and intracellular CD3z activation domain, while the COS RevCAR includes the extracellular peptide epitope E5B9, CD28 HiD, CD28 TMD and intracellular CD28 co-stimulatory domain (ICD).

Considering that the RevTM CEA-IgG4-7B6 showed a superior redirection of RevCAR T-cells compared to the RevTM CEA-7B6, the IgG4-based molecule was selected to target CEA via the SIG RevCAR. To target EpCAM with the COS RevCAR we designed the two novel anti-EpCAM-anti-5B9 RevTMs, either based on a minibody (Mb) or IgG1 Fc backbone (**Figure 5A**). **Figure 5B** illustrates that both RevTMs include the V_H_ and V_L_ domains of an anti-EpCAM mAb, as well as an scFv specific for the detection of the RevCAR epitope E5B9. The two scFvs are either bound through a hinge and C_H_3 domain (RevTM EpCAM-Mb-5B9) or by hinge, C_H_2 and C_H_3 domains (RevTM EpCAM-IgG1-5B9) of a human IgG1 Fc backbone. In order to abolish Fc receptor and complement binding, the later RevTM harbors mutations previously described (54, 58).

Anti-EpCAM RevTMs were produced in a similar way to anti-CEA RevTMs and purified with anti-Strep-Tactin^®^ XT columns. Although the theoretical size of RevTM EpCAM-Mb-5B9 is around 72 kDa and the one from RevTM EpCAM-IgG1-5B9 is 88 kDa, both anti-EpCAM RevTMs run slightly higher on SDS-PAGE gel (**Figures 5C, D**). In addition to the prominent protein bands representing the full-length RevTMs, a few minor bands with higher and lower molecular weights were observed but remained undetected using immunoblotting. These probably represent non-Strep-tagged contaminating proteins derived from the cell culture media. Furthermore, a Strep-tagged band (around 65 kDa) was detected in the immunoblotting of RevTM EpCAM-IgG1-5B9, which might be an aberrant protein conformation or degradation of the RevTM. Both RevTMs EpCAM-Mb-5B6 and EpCAM-IgG1-5B9 were successfully expressed and purified in sufficient yield and quality for pre-clinical functionality studies.

### Binding capability of anti-EpCAM RevTMs and antigen expression of target cells

**Figure 6:**
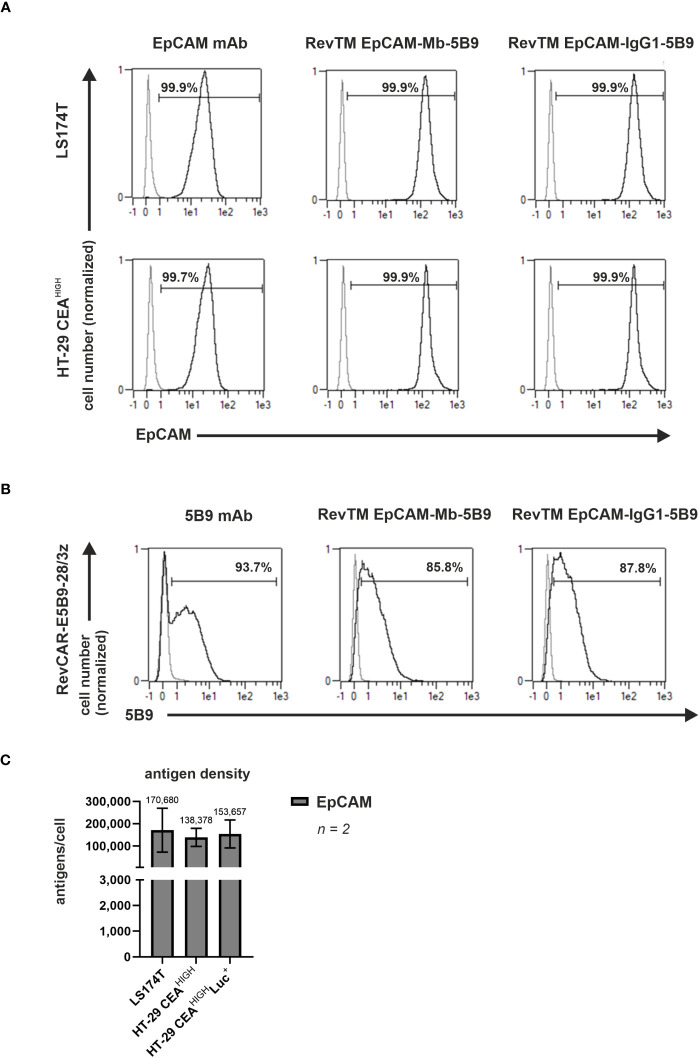
Binding capability of anti-EpCAM RevTMs and EpCAM and CEA density on tumor cell lines. **(A)** LS174T, HT-29 CEA^HIGH^ cells and **(B)** RevCAR-E5B9-28/3z T-cells were stained with the novel anti-EpCAM-anti-5B9 RevTMs and detected with DY-649-conjugated anti-Strep-Tactin^®^ XT mAb. As controls for positive expression, mAbs recognizing EpCAM or E5B9 were included. The commercial EpCAM mAb was detected with AlexaFluor647-conjugated anti-mouse IgG mAb, while the anti-5B9 mAbs with PE-conjugated anti-mouse IgG mAb. Stained cells (black curves) and corresponding controls (gray curves) are displayed as histograms and the percentage of positively stained cells is indicated. Results for one representative binding assay are shown. **(C)** EpCAM density on the surface of target cells was assessed and quantified. Data for two individual experiments are summarized as mean ± SD. Calculated mean value is shown above each column.

Surface expression and density of EpCAM in both CEA-expressing cell models, LS174T and HT-29 CEA^HIGH^ cells was confirmed by staining with a commercial anti-EpCAM mAb (**Figure 6A**) and QIFIKIT (**Figure 6C**).

Moreover, to assess the capacity of the novel anti-EpCAM RevTMs to cross-link EpCAM^+^ cells and RevCAR-E5B9 T-cells surface stainings to LS174T, HT-29 CEA^HIGH^ cells and RevCAR T-cells were performed. As shown in **Figure 6A**, both anti-EpCAM RevTMs strongly bound to LS174T and HT-29 CEA^HIGH^ cells confirming that these RevTMs have an EpCAM binding capability. In addition, **Figure 6B** demonstrates that both novel anti-EpCAM RevTMs also bound to RevCAR-E5B9 T-cells. Overall, these results validate that the anti-EpCAM molecules are able to specifically bind the target antigen of interest as well as the RevCAR-E5B9 T-cells.

### *In vitro* killing potential and cytokine release by redirected Dual-RevCAR T-cells upon dual targeting of CEA and EpCAM according to an AND-gate logic

**Figure 7:**
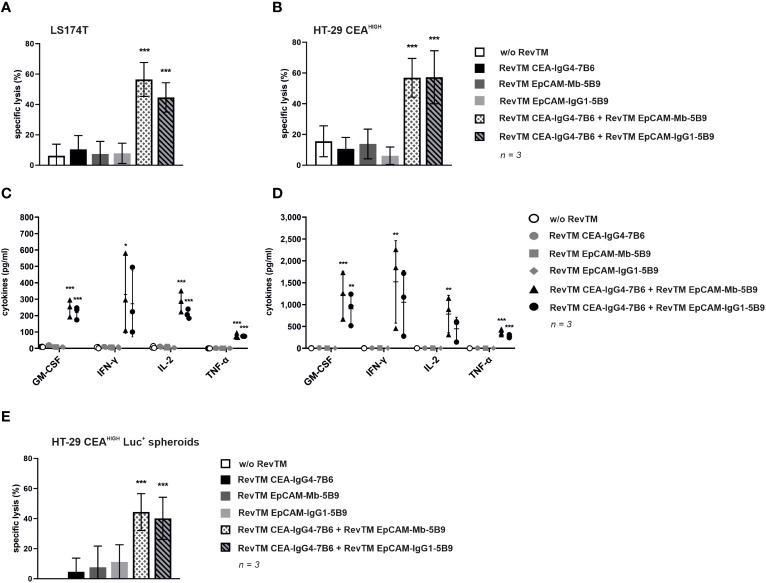
Cytotoxic potential and release of proinflammatory cytokines by redirection of Dual-RevCAR T-cells with anti-EpCAM and anti-CEA RevTMs. **(A,B)** To determine the killing capability of Dual-RevCAR T-cells, standard ^51^Cr-release assays were performed. ^51^Cr-labeled **(A)** LS174T and **(B)** HT-29 CEA^HIGH^ cells were cultured together with Dual-RevCAR T-cells at a 5:1 E:T ratio in the absence, presence or combination of the indicated RevTMs (5 nM) for 48 **(h)** Data for three individual donors were summarized as mean specific lysis ± SD. **(C,D)** Following a similar experimental setup un-labeled **(C)** LS174T and **(D)** HT-29 CEA^HIGH^ cells were co-cultured with Dual-RevCAR T-cells and RevTMs. After 48 h incubation time, cell-free supernatants were collected and analyzed using the MACSPlex Cytokine human kit. Average cytokine concentrations ± SD for three individual donors are displayed. **(E)** To assess the killing capability of 3D cell culture model by Dual-RevCAR T-cells, modified T-cells were cultured together with HT-29 CEA^HIGH^ Luc^+^ spheroids in the absence, presence or combination of RevTMs (5 nM) at a 1:1 E:T for 20 h. Data for three individual donors were summarized as mean specific lysis ± SD. All statistical analyses were performed with one-way ANOVA and Dunnett’s multiple comparison test. Significance with respect to respective controls is shown, p< 0.05 (*), p < 0.01 (**), p < 0.001 (***).

In order to evaluate that only the simultaneous targeting in the presence of anti-CEA and anti-EpCAM RevTMs triggers a full activation and cytotoxic potential of the Dual-RevCAR T-cells upon their encounter with CEA^+^ EpCAM^+^ cancer cells, cytotoxic assays were performed. For this, cancer target cells were cultured together with Dual-RevCAR T-cells in the absence, presence or combination of RevTMs for 48 h. As shown in **Figure 7**, only the simultaneous presence of RevTM CEA-IgG4-7B6 and either RevTM EpCAM-Mb-5B9 or RevTM EpCAM-IgG1-5B9 caused a significant killing of (A) LS174T or (B) HT-29 CEA^HIGH^ cells by redirected Dual-RevCAR T-cells. Activation driven by the combination of anti-CEA and anti-EpCAM RevTMs is also reflected by the significant expression of the activation marker CD69 on Dual-RevCAR T-cells (**Supplementary Figure 2**). It is important to note that both anti-EpCAM RevTMs combined with RevTM CEA-IgG4-7B6 promoted a similar killing and activation. In addition, absence or individual presence of RevTMs induced poor activation and negligible killing activity by the Dual-RevCAR T-cells, which confirms that the full activation of the modified T-cells is dependent on the simultaneous presence of the RevTMs and triggering of both CD3 and CD28 signals. Moreover, **Supplementary Figure 3** validates that cancer cells lacking both target antigens will be spared by Dual-RevCAR T-cells even when cultured with both RevTMs. Altogether, data prove the AND-gate feature of the Dual-RevCAR system targeting CEA and EpCAM on double positive cancer cells.

To monitor the cytokine release pattern, modified T-cells were cultured with LS174T and HT-29 CEA^HIGH^ cells in the absence, presence or combination of RevTMs. After 48 h incubation, cell-free supernatant was analyzed with the MACSPlex Cytokine human kit. As summarized in **Figures 7C, D**, Dual-RevCAR T-cells secreted increased amounts of GM-CSF, IFN-γ, IL-2 and TNF-α only upon engaging CEA^+^ EpCAM^+^ cancer cells upon simultaneous presence of anti-CEA and anti-EpCAM RevTMs. The combination of both RevTMs promoted a higher secretion of cytokines when Dual-RevCAR T-cells were cultured together with HT-29 CEA^HIGH^ cells compared to culture with LS174T cells, which mirrors the activation profile discrepancy of both conditions (**Supplementary Figure 2**). Importantly, no increase in cytokine levels was detected on the conditions testing individual RevTMs alone or no RevTMs, which once again validates the RevTM-dependency and AND-gate logic of Dual-RevCAR T-cells.

Aiming to better mimic a 3D tumor structure, HT-29 CEA^HIGH^ Luc^+^ spheroids were formed and co-cultured with Dual-RevCAR T-cells in the absence, presence and combination of RevTMs. **Figure 7E** demonstrates that the killing of a 3D model by Dual-RevCAR T-cells corroborates the results observed using monolayer cultures. As expected, HT-29 CEA^HIGH^ Luc^+^ spheroids were recognized and eliminated only when anti-CEA and anti-EpCAM RevTMs were combined with Dual-RevCAR T-cells. Similar to the observations from monolayer cytotoxic assays, no significant difference was identified between the killing effect promoted by the different anti-EpCAM RevTM formats when combined with RevTM CEA-IgG4-7B6. Additionally, absence or individual presence of RevTMs caused no significant killing of the spheroids.

Altogether, the results confirm that 2D monolayers and 3D spheroids derived from colon cancer cell lines can be targeted by combined CEA and EpCAM recognition following a true AND-gate logic with the Dual-RevCAR system.

### *In vivo* inhibition of tumor growth by Dual-RevCAR T-cells upon dual targeting of CEA and EpCAM according to an AND-gate logic

**Figure 8:**
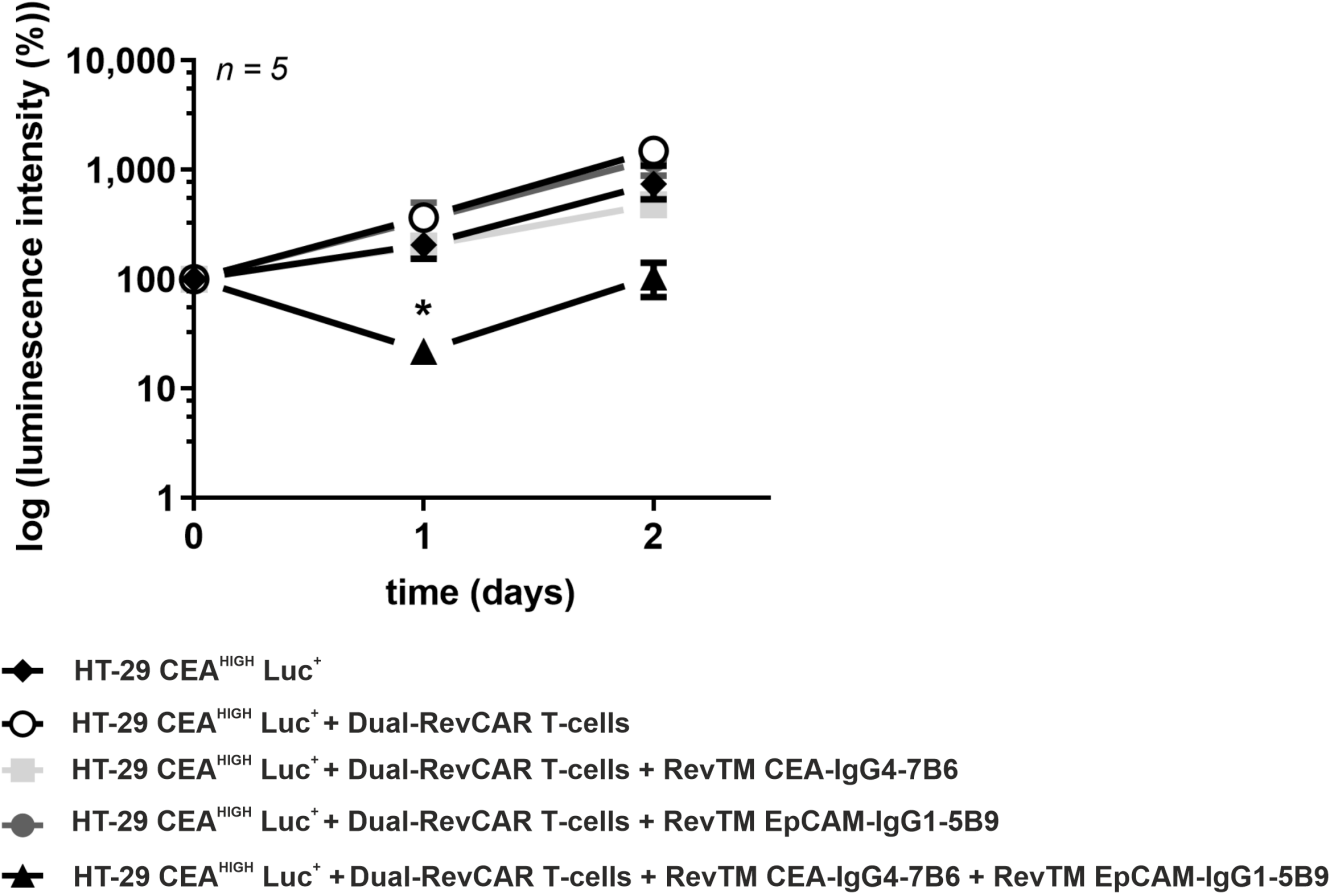
*In vivo* tumor growth impairment by redirected Dual-RevCAR T-cells with anti-CEA and anti-EpCAM RevTMs. Female NXG mice were injected with HT-29 CEA^HIGH^ Luc^+^ cells alone, together with Dual-RevCAR T-cells in the absence, presence or combination RevTM CEA-IgG4-7B6 and RevTM EpCAM-IgG1-5B9. Tumor growth was monitored by bioluminescence imaging for 2 days. A quantitative analysis was performed and values were normalized to the initial measurement performed at the day of injection (Day 0). Data are presented as group mean ± SD. For statistical analysis two-way ANOVA with Dunnett’s comparison test was performed. Significant differences between control group HT-29 CEA^HIGH^ Luc^+^ + Dual-RevCAR T-cells against the experimental group including both RevTMs is showed with asterisk (*). p < 0.05 (*).

The *in vivo* anti-tumor activity of Dual-RevCAR T-cells redirected via combination of anti-CEA and anti-EpCAM RevTMs was studied. Five groups of female NXG mice were randomly formed. Control groups received HT-29 CEA^HIGH^ Luc^+^ cells alone or in combination with Dual-RevCAR T-cells. The other groups received tumor cells, Dual-RevCAR T-cells together with either RevTM CEA-IgG4-7B6, RevTM EpCAM-IgG1-5B9 or a combination of both RevTMs. Tumor growth was monitored by bioluminescence imaging. Quantitative results shown in **Figure 8** indicate that only the treatment with administration of both RevTM CEA-IgG4-7B6 and RevTM EpCAM-IgG1-5B9 inhibits tumor growth compared to the control group that received HT-29 CEA^HIGH^ Luc^+^ together with Dual-RevCAR T-cells. Although not all tumor cells could be eradicated completely, the observed anti-tumor cell effect was significant directly after the co-injection. Still remaining tumor cells slightly grew out again afterwards most probably because one or both RevTMs were not sufficiently available anymore. Overall we conclude, the initial *in vivo* proof-of-concept data corroborate the true AND-gate logic of the Dual-RevCAR T-cells observed during *in vitro* experiments.

## Discussion

One major problem related to targeting of solid tumors with CAR T-cells is the lack of selectively expressed tumor targets with the consequence that healthy tissues will also be attacked by CAR T-cells, which in some cases can even become life-threatening (15). The outcome of various clinical trials demonstrate that these are not isolated cases and reiterates the need to steer the activity of the modified T-cells after infusion into the patient (16, 59, 60). Aiming to improve safety of CAR T-cell therapy, our group developed the modular adaptor platforms UniCAR and RevCAR, in which the T-cells can be controlled via tumor-specific TMs. The RevCAR platform was developed for two reasons: (i) to reduce the risk of tonic signaling caused by oligomerization of light and heavy chains of neighboring CARs on the surface of CAR T cells and (ii) to reduce the size of CAR genes allowing us to simultaneously transduce at least two CARs into the same T-cell with a single vector for dual targeting according to AND-gate logic.

In view of the prevalence and mortality of CRC, this work explores the possibility of adapting the RevCAR system as a more specific and safer alternative treatment for this type of cancer. As off-tumor toxicities are a common trait when targeting solid tumors with CAR T-cell approaches, we wanted to explore the feasibility of a dual targeting approach following AND-gated logic for CRC, meaning only if two target antigens are present on the same cell the effector CAR T-cell should be activated. As a potential pair of target antigens we selected the two TAAs CEA and EpCAM as both antigens are known to be overexpressed in malignant CRC tissues (6, 11, 13, 14). For AND-gated targeting, we used CEA as target for the signaling receptor and EpCAM for the receptor providing co-stimulation. The reasons for this decision were that CEA is overexpressed through most CRC patient materials, although it is also expressed on healthy glandular epithelia (6). However, its polar distribution might turn it into a relatively safe antigen for targeting, considering that healthy cells expose CEA only in the luminal side, not available for CAR T-cell recognition (8). In contrast, malignant cells lose this polar CEA distribution and express the antigen over the entire cell surface. This probably explains the successful tumor shrinkage promoted by anti-CEA CAR T-cells observed in a clinical trial (NCT01373047), in which patients did not experience severe secondary effects (61). Nevertheless, injury of healthy tissue may result in depolarized distribution of this antigen (8). For EpCAM, however, its normal expression on epithelium has been proven to be a safety issue if targeted individually (9, 10, 18). Therefore, of the two targets CEA appeared to be the “safer” target, while EpCAM may have a higher risk of off-target activation when used for the signaling RevCAR. On the other hand, EpCAM is likely available for co-stimulation on most tumor tissues while the presence on healthy tissues should not be harmful due to lack of primary signaling through the CAR as long as CEA is not expressed. It is unlikely that CEA is simultaneously accessible due to luminal expression of CEA (8) and the basolateral expression of EpCAM in, e.g., normal gut epithelium (52). Thereby, aiming to achieve a true AND-gate targeting that can potentially diminish off-target toxicities the strategy followed here was to have the SIG RevCAR binding to the more delimited expressed antigen (CEA) and the COS RevCAR binding to the more broadly found antigen (EpCAM).

Following this concept, in a first step we generated the novel anti-CEA RevTMs for targeting CEA^+^ cancer cells using a second-generation CAR setting. Two RevTM formats that differ with respect to their molecular size, backbone structure and binding valency were constructed: a monomeric small sized scFv-based RevTM and a homodimeric size-extended IgG4-based RevTM. We envision that the small format can be infused into the patient at early stages of clinical treatment, when a faster tuning of the T-cells is needed in case severe side effects occur, while the administration of the IgG-like RevTM at later stages allows us to increase the time intervals between the infusions for the convenience of the patient (24, 32, 48).

Both, RevTM CEA-7B6 and RevTM CEA-IgG4-7B6 showed binding capability towards CEA^+^ cancer cells and RevCAR-E7B6 T-cells, in addition to induce specific lysis of CEA^+^ target cells accompanied by secretion of proinflammatory cytokines. Direct comparison of both structures revealed that RevTM CEA-IgG4-7B6 bound more efficiently to cancer cells than the scFv-based molecule (K_D_ = 35.6 nM vs K_D_ = 175.2 nM, respectively). Similarly, RevTM CEA-IgG4-7B6 appears to promote a more efficient killing of CEA^+^ cancer cells, indicated by EC_50_ values in the picomolar range, in comparison with RevTM CEA-7B6, whose EC_50_ values belong to the nanomolar range. Moreover, IgG4-based RevTM induces a higher secretion of cytokines. This aligns with previous observations demonstrating that the increase of binding moieties of UniCAR TMs or RevTMs led to a superior affinity and killing efficiency against target cells in relation to a monomeric structure (24, 34). Apparently, these molecules allow a more stable and prolonged cross-linking of the target and effector cells, which enables a stronger cytotoxicity. As the dimeric RevTM CEA-IgG4-7B6 has two RevCAR binding arms, it bears the possibility to activate RevCAR T-cells by cross-linking two RevCARs in the absence of target-expressing tumor cells similar to the CAR signaling effect observed for other bivalent soluble molecules (62). We have seen no evidence that the RevTM CEA-IgG4-7B6 is able to sufficiently activate RevCAR-E7B6 T-cells and trigger cytokine release in the absence of CEA-positive cancer cells (data not shown). Overall, our data confirms that the activation of RevCAR-E7B6 T-cells depends exclusively on their cross-linkage with CEA-expressing cancer cells via the respective RevTM in a TM- and target-specific manner.

As adaptor CAR T-cells represent a safer approach by regulating T-cell activity, the described RevCAR system targeting CEA^+^ cancer cells could already be applicable for the treatment of patients. However, to further decrease the risk of “on-target, off-tumor” toxicity, we adapted our Dual-RevCAR platform for CRC following the idea of AND-gated targeting. For this reason, further novel RevTMs targeting EpCAM had to be developed to target the co-stimulatory RevCAR receptor. We show that only the combination of RevTM CEA-IgG4-7B6 with RevTM EpCAM-Mb-5B9 or RevTM EpCAM-IgG1-5B9 induces the activation of Dual-RevCAR T-cells and the subsequent secretion of proinflammatory cytokines and killing of target cells. As expected, the stimulation of only one of the RevCAR type is not enough to result in a full activation of the modified T-cells neither specific killing of target. This is observed when only one of the RevTMs is administered or when the target cells express only one of the target antigens. Importantly, if the cells do not express an antigen, the Dual-RevCAR T-cells did not induce killing. The observations were not only obtained in monolayer cultures but also with 3D cultures and during proof-of-concept *in vivo* experiments. To prolong an initially observed tumor suppression *in vivo* a permanent or repeated infusion of RevTMs would be necessary. The results prove that the true AND-gate feature of the Dual-RevCAR system represents a more specific and safer approach by reducing the risk of off-tumor targeting. Moreover, the present study demonstrated that Dual-RevCAR T-cells remain inert when no RevTMs are administered, which validates once more the efficient switch on-off possibility of our adaptor CAR platforms. No differences were observed between the effects produced by the two different anti-EpCAM RevTM structures, probably because both have the same number of binding moieties and their size difference is not very pronounced. Here, it was observed that an IgG4-based RevTM in combination with a Mb- or IgG1 Fc-based RevTM mediated a true AND-gate targeting of CEA^+^ EpCAM^+^ cells. Considering the fact that in earlier Dual-RevCAR studies (22, 23), in which we analyzed bispecific scFv-based RevTM structures, we here demonstrated that the Dual-RevCAR platform also performs well when different RevTM formats are combined. This presents the opportunity to create and combine diverse RevTM structures, taking advantage of their individual pharmacological properties and enabling a more precise customization of the RevCAR system in response to the patient’s individual reactions.

## Conclusion

In conclusion, we demonstrated the preclinical effective targeting of CRC cells with the RevCAR system, providing an increased degree of safety due to its switch on-off feature. In addition, we developed novel RevTMs for an adaptor AND-gate combinatorial targeting approach, with the potential to further improve the specific recognition of CEA^+^ EpCAM^+^ cancer cells. In contrast to traditional CAR T-cell therapies, an AND-gate approach based on adaptor CARs not only has the potential to reduce the risk of off-tumor toxicities but also offers a more controllable treatment alternative.

## Data availability statement

The datasets presented in this article are not readily available because material and reagents generated in this study will be made available upon request and settlement of a Material Transfer Agreement. Requests to access the datasets should be directed to Michael Bachmann, m.bachmann@hzdr.de, Anja Feldmann, a.feldmann@hzdr.de.

## Ethics statement

The studies involving human T-cells were approved by the Medical Faculty Carl Gustav Carus, Technical University Dresden, Germany (EK138042014). The studies were conducted in accordance with the local legislation and institutional requirements. The animal study was approved by German Regulations for Animal Welfare. The study was conducted in accordance with the local legislation and institutional requirements (DD24.1-5131/449/67 and DD24.1-5131/449/49).

## Author contributions

KS: Formal analysis, Investigation, Methodology, Visualization, Writing – original draft, Writing – review & editing. LL: Conceptualization, Formal analysis, Methodology, Supervision, Visualization, Writing – original draft, Writing – review & editing. TB: Investigation, Writing – review & editing. CA: Writing – review & editing. AK: Writing – review & editing. NM: Writing – review & editing. LD: Writing – review & editing. LH: Writing – review & editing. HS: Writing – review & editing. EC: Writing – review & editing. MM: Writing – review & editing. CD: Writing – review & editing. HA: Resources, Writing – review & editing. FM: Resources, Writing – review & editing. MB: Conceptualization, Funding acquisition, Resources, Writing – review & editing. AF: Conceptualization, Formal analysis, Resources, Supervision, Writing – original draft, Writing – review & editing.
