# Supplementary material for: Targeting colorectal cancer cells using AND-gated adaptor RevCAR T-cells
Source: Front Immunol. 2023 Dec 15;14:1302354. doi: 10.3389/fimmu.2023.1302354 (PMC10758449; doi:10.3389/fimmu.2023.1302354)
Supplement: Supplementary file 1 [file DataSheet_1.pdf]

Supplementary Material

Targeting colorectal cancer cells using AND-gated Adaptor RevCAR T-cells

Karla E. G. Soto[1†], Liliana R. Loureiro[1†], Tabea Bartsch[1], Claudia Arndt[1,2], Alexandra Kegler[1], Nicola Mitwasi[1], Laura Drewitz[1], Lydia Hoffmann[1], Haidy A. Saleh[1], Eugenia Crespo[1], Maria Mehnert[1,2], Cansu Daglar[1], Hinrich Abken[3], Frank Momburg[4,5], Michael Bachmann[1,6,7,8*] and Anja Feldmann[1,6,7,8*]

*** Correspondence:**

Michael Bachmann and Anja Feldmann
m.bachmann@hzdr.de and a.feldmann@hzdr.de

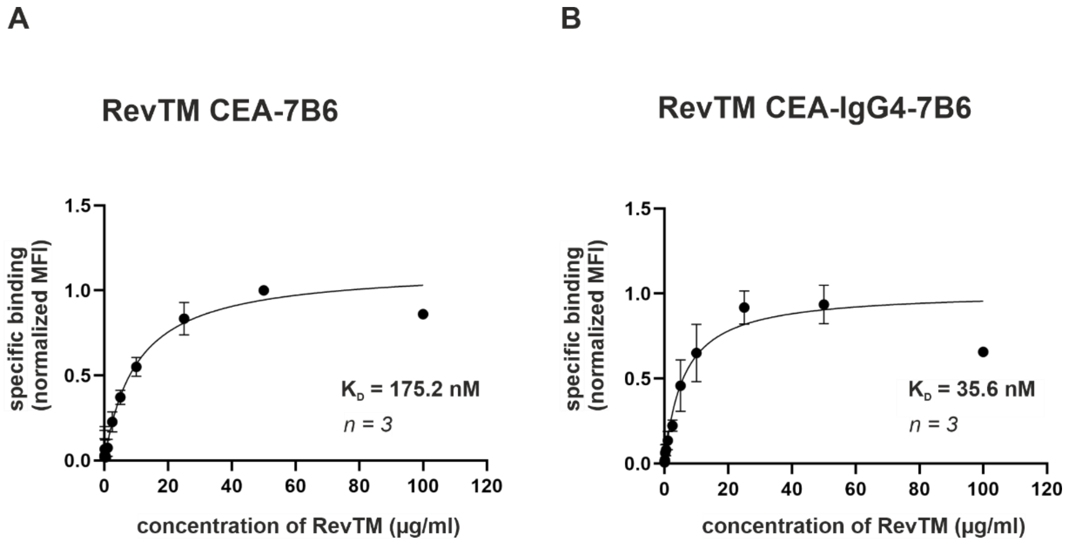

Supplementary Figure 1. K_D values of anti-CEA RevTMs. HT-29 CEA[HIGH] cells were stained with increasing concentrations of (A) RevTM CEA-7B6 and (B) RevTM CEA-IgG4-7B6 and detected with PE-conjugated anti-His mAb. K_D values were calculated from the generated binding curve. Data are presented as median fluorescence intensity (MFI) average values ± SD of three independent measurements.

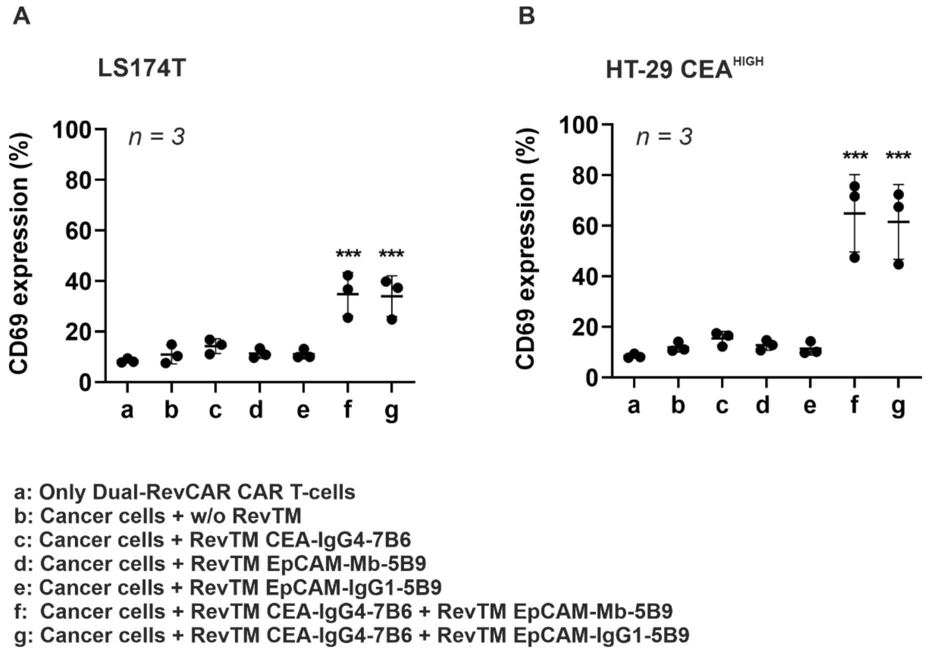

Supplementary Figure 2. Activation of Dual-RevCAR T-cells upon dual targeting of CEA and EpCAM according to an AND-gate logic. Dual-RevCAR T-cells were cultured with (A) LS174T or (B) HT-29 CEA^{HIGH} cells under different conditions (a-g, as displayed in the figure) for 48 h. After the incubation time, cells were recovered, stained and analyzed with flow cytometry. The expression of CD69 was evaluated to examine the activation of Dual-RevCAR T-cells. Percentage CD69-expression of three individual donors is pictured as single points; in addition to mean ± SD. Statistical significance with respective controls was determined using one-way ANOVA with Dunnett's multiple-comparison test and it is indicated with asterisks (*); $p < 0.001$ (***).

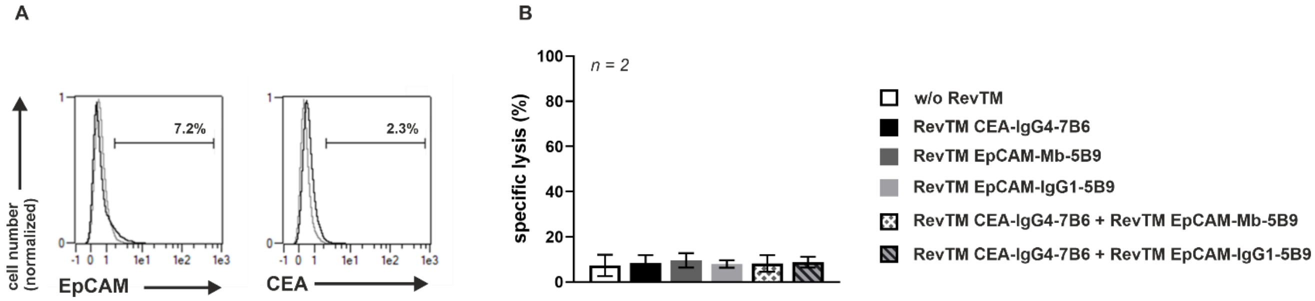

Supplementary Figure 3. Dual-target-specific effect of Dual-RevCAR T-cells. (A) MDA-MB-231 cells (CEA^- $EpCAM^-$) were stained with commercial anti-CEA and anti-EpCAM mAbs and detected with AlexaFluor647-conjugated anti-mouse IgG mAb. As negative control, an isotype control IgG1 was included. Stained cells (black curves) and corresponding controls (gray curves) are displayed as histograms and the percentage of positively stained cells is shown. Results for one representative binding assay are shown. ^{51}Cr-labeled (B) MDA-MB-231 cells were cultured together with Dual-RevCAR T-cells at a 5:1 E:T ratio in the absence, presence or combination of the indicated RevTMs (5 nM) for 48 h. Triplicates for each condition were included. Data for two individual donors were summarized as mean specific lysis ± SD.
